# The Metabolic Regulation of Amino Acid Synthesis Counteracts Reactive Nitrogen Stress via *Aspergillus nidulans* Cross-Pathway Control

**DOI:** 10.3390/jof10010058

**Published:** 2024-01-10

**Authors:** Madoka Amahisa, Madoka Tsukagoshi, Chihiro Kadooka, Shunsuke Masuo, Norio Takeshita, Yuki Doi, Hiroshi Takagi, Naoki Takaya

**Affiliations:** 1 Microbiology Research Center for Sustainability, Faculty of Life and Environmental Sciences, University of Tsukuba, 1-1-1 Tennodai, Tsukuba 305-8572, Japan; s2220973@u.tsukuba.ac.jp (M.A.); kadooka@bio.sojo-u.ac.jp (C.K.); masuo.shunsuke.fp@u.tsukuba.ac.jp (S.M.); takeshita.norio.gf@u.tsukuba.ac.jp (N.T.); doi.yuki.ge@u.tsukuba.ac.jp (Y.D.); 2 Institute for Research Initiatives, Nara Institute of Science and Technology, 8916-5 Takayama, Ikoma 630-0192, Japan; hiro@bs.naist.jp

**Keywords:** nitric oxide, stress, reactive species, amino acid, starvation

## Abstract

Nitric oxide (NO) is a natural reactive nitrogen species (RNS) that alters proteins, DNA, and lipids and damages biological activities. Although microorganisms respond to and detoxify NO, the regulation of the cellular metabolic mechanisms that cause cells to tolerate RNS toxicity is not completely understood. We found that the proline and arginine auxotrophic *proA5* and *argB2* mutants of the fungus *Aspergillus nidulans* require more arginine and proline for normal growth under RNS stress that starves cells by accumulating fewer amino acids. Fungal transcriptomes indicated that RNS stress upregulates the expression of the biosynthetic genes required for global amino acids, including proline and arginine. A mutant of the gene disruptant, *cpcA*, which encodes the transcriptional regulation of the cross-pathway control of general amino acid synthesis, did not induce these genes, and cells accumulated fewer amino acids under RNS stress. These results indicated a novel function of CpcA in the cellular response to RNS stress, which is mediated through amino acid starvation and induces the transcription of genes for general amino acid synthesis. Since CpcA also controls organic acid biosynthesis, impaired intermediates of such biosynthesis might starve cells of amino acids. These findings revealed the importance of the mechanism regulating amino acid homeostasis for fungal responses to and survival under RNS stress.

## 1. Introduction

The most investigated reactive nitrogen species (RNS) is gaseous nitric oxide (NO), which many organisms produce and use for signaling. Mammalian NO synthase uses arginine as a substrate to synthesize NO and regulate smooth muscle vasodilation as well as other processes [1,2]. Bacterial and fungal NO synthase-like enzymes synthesize NO [3,4], but the full extent of their physiological roles is unknown [5]. Bacterial and fungal denitrification mechanisms produce NO as an intermediate of nitrate reduction [6]. Some microorganisms utilize nitrate as a nutrient, and its metabolic intermediate, nitrite, is a source of RNS nitrosonium cations under physiological, especially acidic conditions [7]. Fungal nitrate reductase produces NO independently of its primary role in nitrogen assimilation [8]. Thus, many organisms synthesize and utilize NO to maintain normal physiological functions.

Microbial mechanisms that enable adaptation to NO have been suggested because high concentrations of NO and its derivatives are highly cytotoxic. Nitric oxide chemically reacts with thiol groups on cysteine residues in proteins and with cofactors, such as iron-sulfur clusters, heme, and metal ions in enzymes, and thus can inhibit intracellular metabolism. Tolerance mechanisms against NO have been investigated using *Aspergillus nidulans*, which is a popular model for studies of the molecular biology of filamentous fungi. Exposing *A. nidulans* to RNS causes aconitase inhibition in the tricarboxylic acid (TCA) cycle and in mitochondrial respiration [9]. Microorganisms have developed protective mechanisms against RNS. Fungal and bacterial flavohemoglobin is a NO dioxygenase that converts NO and O_2_ to less toxic nitrate [10,11,12]. Filamentous fungi produce nitrosothionein (NtpA), which, together with thioredoxin reductase, detoxifies NO via its *S*-nitroso-form intermediates [13], and the NO reductase cytochrome P450nor converts NO to less toxic nitrous oxide (N_2_O). Therefore, the chemical properties of NO described above indicate that, on the one hand, NO levels in cells must be controlled, but on the other hand, NO influences global cellular metabolism. Besides NO detoxification mechanisms, the mechanism(s) of adaptation to global metabolic disruption caused by NO remains unknown.

Amino acid homeostasis is maintained in yeast and filamentous fungi via general carbon/nitrogen and cross-pathway control mechanisms, respectively. The bZIP family of transcription factors that are conserved among fungi activates the expression of genes encoding proteins that biosynthesize amino acids upon their depletion. *Saccharomyces cerevisiae* responds to amino acid starvation and increases the intracellular levels of the bZIP general control transcription factor (Gcn4p) [14]. Gcn4p binds to a specific DNA sequence motif in target gene promoters and activates gene expression. In addition to amino acids, Gcn4p also regulates genes encoding purine biosynthesis enzymes. The cross-pathway control gene (*cpcA*) in *A. nidulans* encodes a bZIP transcription factor with an amino acid sequence that is 40% identical to those of *S. cerevisiae* Gcn4p and *Neurospora crassa* Cpc-1 [15,16,17]. Both transcription factors respond to amino acid starvation induced through the amino acid biosynthesis inhibitor 3-aminotriazole [18]. These sequence and functional similarities indicate a CpcA function that regulates global gene expression related to amino acid synthesis, but the target genes and regulation mechanisms of *A. nidulans* CpcA are only partly characterized [19].

We screened *A. nidulans* for genes that allow fungi to grow under RNS stress by introducing multiple copies of fungal genes and identified a *proC* paralog. Consistent with this finding, the fungus under RNS stress accumulated fewer intracellular amino acids and required abundant proline and arginine for normal growth. We found that CpcA upregulated the transcription of genes for synthesizing amino acids and maintained normal intracellular amino acid levels under RNS stress. These findings revealed that a fungal cross-pathway control mechanism that regulates NO-dependent gene expression is a metabolic adaptive strategy to protect against global metabolic disruption caused by RNS.

## 2. Materials and Methods

### 2.1. Strains, Culture, and Media

Table 1 shows the *A. nidulans* strains used in this study. Host strains for transformation were *A. nidulans* YMS9 and TN02A3. Strains A45, A89, and TN02A3 (Fungal Genetic Stock Center, University of Kansas Medical Center, Kansas City, KS, USA) [20] were maintained in minimal medium (MM) containing 1% glucose, 6 g L^−1^ NaNO_3_, 10 mM potassium phosphate, 7 mM KCl, 2 mM MgSO_4_, and 1 mL L^−1^ trace element stock solution [21] (pH 6.5). Auxotrophic mutants were maintained in biotin, pyridoxine (0.4 mg L^−1^ each), uridine plus uracil (4 and 5 mM), proline, and arginine (1 mM each). We assayed RNS stress in minimal NO medium (MMN) containing 1% glucose, 10 mM ammonium tartrate, 20 mM potassium phosphate, 7 mM KCl, 2 mM MgSO_4_, and 1 mL L^−1^ trace element stock solution (pH 5.5). Conidia (2.0 × 10^7^) were incubated at 37 °C for 18 h at 120 rpm in 500-mL Erlenmeyer flasks containing 100 mL of MMN with final NaNO_2_ concentrations of 1 or 10 mM. Serial dilutions of conidia were spotted onto solid 1.5% agar and incubated at 37 °C for 2 days. Thereafter, we analyzed the colony morphology and growth to determine RNS tolerance.

### 2.2. Screening the RNS Tolerance Genes

Strain YMS9 was transformed with the pRG3-AMA1-NotI WT library (Fungal Genetic Stock Center) [22] and replicated in MMN containing 10 mM NaNO_2_ (pH 5.5). The growth was monitored at 37 °C for 3 days, and then the total DNA was introduced into *Escherichia coli* DH5α to generate *E. coli* transformants as described in [9]. Plasmids were recovered from the bacterial transformants, and the nucleotide sequences of the inserted fragments were determined using the primers oMN33_3 and oMN33_5 (Supplementary Table S2). The fragments of inserted DNA were amplified via PCR. The specific primer sets AN4354, AN4354 + d500, AN4355, and AN4355 + d500 were cloned into pRG3-AMA1 digested with *Kpn*I [22]. Then, strain YMS9 protoplasts were transformed using recombinant plasmids. We then identified AN4355 as the RNS tolerance gene by analyzing the transformant growth under RNS stress. 

### 2.3. Determination of Amino Acids and Cell Weight

*Aspergillus nidulans* strains were stirred at 120 rpm in MMN medium (pH 5.5) for 18 h 37 °C, followed by final concentrations of 1 or 10 mM sodium nitrite for 3 h. Mycelia were collected via filtration, and portions (~0.1 g wet weight) frozen in liquid nitrogen were pulverized by running two cycles at 3000 rpm for 5 s in a Multi-beads Shocker^®^ MB3200 (Yasui Kikai, Osaka, Japan). The homogenates were suspended in 1 mL of 90% methanol containing 5 μM 2-morpholinoethanesulfonic acid, vortex-mixed for 3 min, and centrifuged at 15,000 rpm at 4 °C for 15 min. The supernatants were lyophilized and solubilized in water. The intracellular amino and other organic acids (OAs) were fractionated using an LCMS-8045 liquid chromatography tandem mass spectrometer (Shimadzu Co., Kyoto, Japan) equipped with a 150 × 2.1-mm Discovery HS F5 (Supelco Inc., Bellefonte, PA, USA) with 3-μm particles and 120-Å pores. The mobile phase was 0.1% formic acid (solvent A) and acetonitrile containing 0.1% formic acid (solvent B). The metabolites were eluted using the following gradient profile: 0% B, 0–2 min; 0%–25% B, 2–5 min; 25%–35% B, 5–11 min; 35–95% B, 11–15 min; 95% B, 15–20 min, and 0% B, 20.1– 25 min. The operating parameters of the mass spectrometer were as follows: capillary voltage, 4.5 kV; desolvation line, 250 °C; heat block, 400 °C; nebulizer nitrogen gas, 3 L min^−1^; drying gas, 10 L min^−1^. The data were analyzed using LabSolutions LCMS software v.5.96 with the LC/MS/MS Method Package for Primary Metabolites v.2 (Shimadzu Co.). 

### 2.4. Gene Disruption

We constructed gene disruptants using standard double-crossover methods. The 5′- and 3′ untranslated regions of *cpcA* and the *pyrG* gene (transformation marker) were amplified via PCR using the specific primer sets cpcA_up F/R, cpcA_dw F/R, and pyrG F/R to construct a *cpcA* disruption cassette. The amplicons were fused via PCR using nested primers (cpcA_nested F/R), and then protoplasts of *A. nidulans* YMS9 were transformed using the resulting disruption cassette as described below in Section 2.5. We confirmed *cpcA* gene disruption via PCR using the primer sets cpcA_up F and cpcA_dw R. We similarly disrupted the *proC* paralogs, and the transformation marker gene was *pyrG*. Supplementary Table S2 shows the primers used for gene disruption.

### 2.5. Aspergillus nidulans Transformation

*Aspergillus nidulans* was transformed as described in [23] with slight modifications. The conidia (2.0 × 10^7^) of the host strain were inoculated into 100 mL of yeast extract-glucose (YG) medium containing 1% glucose, 0.5% yeast extract, and 1 mL L^−1^ trace element stock solution in 500 mL volumetric flasks and shaken at 120 rpm for 16 h at 32 °C. The mycelia were collected into 50 mL volumes using an autoclaved funnel with a Miracloth (MilliporeSigma, Burlington, MA, USA) and passed through a 0.20 µm filter. The filtrate enzyme solution (15 mL maleic acid buffer; 50 mM maleic acid, 0.6 M (NH_4_)_2_SO_4_, pH 5.5), 25 mg yatalase (Takara Bio Inc., Kusatsu, Japan), and 7.5 mg lysing enzyme (Sigma) were rotated at 100 rpm for 2 h at 30 °C to release the protoplasts by digesting the cell walls. The protoplasts were washed in 1 mL of solution I (10 mM CaCl_2_, 0.8 M NaCl, 10 mM Tris-HCl, pH 7.5), centrifuged at 1000× *g* for 5 min at 4 °C, and the supernatants were discarded. This process was repeated three times. The protoplasts were brought to a density of 2.0 × 10^8^ cells mL^−1^ in solution I. Then, 0.2 ml volumes of solution II (40% PEG4000, 50 mM CaCl_2_, 50 mM Tris-HCl, pH 7.5) were added and gently mixed by tapping the solution (protoplast solution). A mixture of the disruption cassette and 200 µL of the protoplast solution was placed on ice for 40 min. Solution II (1 mL) was added, mixed, and placed at room temperature for 15 min. Soft agar selective medium (5 mL of 0.8% agar containing 0.6 M KCl and pyridoxine) was added, and the mixture was quickly layered on agar plate medium (1.5% agar containing 0.6 M KCl and pyridoxine) and incubated at 37 °C for 2–3 days. The spores from the colonies were transplanted and incubated at 37 °C for 2 days, then the surviving colonies were designated as transformants, and gene disruption was confirmed via PCR as described above.

### 2.6. Quantitative (q)PCR

The *A. nidulans* strains were cultured in liquid MMN (pH 5.5) at 37 °C and rotated at 120 rpm for 18 h, then incubated with acidified nitrite as described above for 3 h. The total RNA was recovered from the mycelia using RNeasy Plant mini kits (Qiagen, Hilden, Germany) and reverse transcribed using Reverse Transcriptase M-MLV (Takara Bio Inc.). Specific sequences were amplified via qPCR using IQ SYBR Green Supermix (Bio-Rad Laboratories Inc., Hercules, CA, USA) with cDNA as the template as described by the manufacturer. The target gene expression was calculated relative to that of actin (*actA*). Supplementary Table S2 shows the primer sets for qPCR.

### 2.7. Sequencing mRNA

*A. nidulans* strains were incubated in MMN (pH 5.5) at 37 °C for 18 h, followed by acidified nitrite as described above for 3 h, and then the total RNA in the mycelia was extracted using RNAiso plus (Takara Bio Inc.). The messenger RNA was sequenced at the Department of Sports Medicine (Open Facility Network Office, University of Tsukuba, Tsukuba, Japan). A cDNA library prepared as described in [24] was sequenced using a Next Seq 500 system (Illumina Inc., San Diego, CA, USA). We mapped FASTQ files to the *A. nidulans* genome (https://fungidb.org/fungidb/app/record/dataset/DS_4b35c88aa1, accessed on 6 January 2023) and the gene expression was evaluated as transcripts per kilobase million (TPM) using CLC Genomics Workbench 20.0.4 (Qiagen). The sequence data and experimental information were deposited in the DNA data bank of Japan (DDBJ; https://www.ddbj.nig.ac.jp/index-e.html, accessed on 21 April 2022), and the accession ID for BioProject is PRJDB15667.

### 2.8. Informatics Analysis

Gene Ontology (GO) enrichment was analyzed using Fungi DB (https://fungidb.org/fungidb/app/, accessed on 14 July 2022). The binding sites for Gcn4p were identified by analyzing the nucleotide sequences upstream of the ATG codon (600 nt each) using YEAst Search for Transcriptional Regulators and Consensus Tracking (YEASTRACT; http://www.yeastract.com/formtfsbindingsites.php, accessed on 15 July 2022). Enrichment of the reported transcription factor binding sites was analyzed using SEA (https://meme-suite.org/meme/tools/sea, accessed on 15 July 2022).

### 2.9. Enzyme Activity

Pyruvate dehydrogenase activity was measured in 100 μL reactions containing cell-free extract (20 μL) as described in [25]. Aconitase activity was measured as described in [9,26] with some modifications; the reactions (100 μL) contained 50 mM Tris-HCl (pH 8.0), 1 mM *cis*-aconitate, 1 mM MgCl_2_, 2 mM NADP^+^, 1 unit mL^−1^ isocitrate dehydrogenase (Fujifilm Wako Pure Chemical Corp., Osaka, Japan), and 20 μL of cell-free extracts. The absorbance at 340 nm was monitored using NADPH and changes were calculated using a molar extinction coefficient of 6.22 mM^−1^ cm^−1^. 

## 3. Results

### 3.1. Proline and Arginine Are Essential for RNS Tolerance

**Figure 1:**
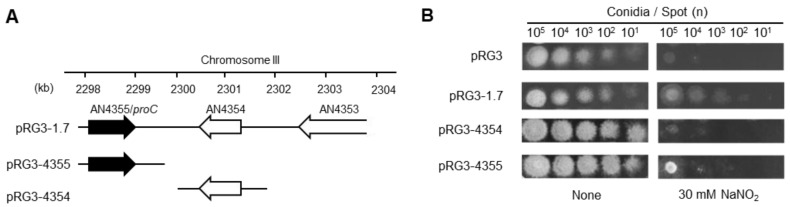
*Aspergillus nidulans proC* gene tolerates NO. (**A**) Insertion of pRG3-1.7 and its derivatives. (**B**) Conidia incubated on MMN agar without or with 30 mM NaNO_2_ (pH 5.5) generated 1 × 10^1^–10^5^ colonies from YMS9 (parental strain), harboring indicated plasmids at 37 °C for 48 h. MMN, minimal nitric oxide medium; NaNO_2_, sodium nitrite; NO, nitric oxide.

We transformed *A. nidulans* protoplasts with a gene library constructed in the multi-copy plasmid vector pRG3 [22] and identified the upregulated genes that conferred growth tolerance to RNS stress. Acidified nitrite (pH 5.5) as a RNS donor and sole nitrogen source [9,13] has led to the identification of a nitrite reductase gene (*niiA*) that decreases the cellular levels of nitrite and results in toxic RNS [9]. To avoid this effect, we added ammonium to the culture medium to suppress *niiA* expression [27] and identify more genes associated with RNS tolerance. We isolated 31 clones that could grow under RNS stress. Plasmid extraction and insert sequencing identified a region containing two genes that potentially mediate RNS tolerance. Respective genes were introduced to the parental *A. nidulans* strain using the pRG3 vector. Then, we analyzed the growth of the transformants upon which *AN4355* confers RNS tolerance (Figure 1). 

**Figure 2:**
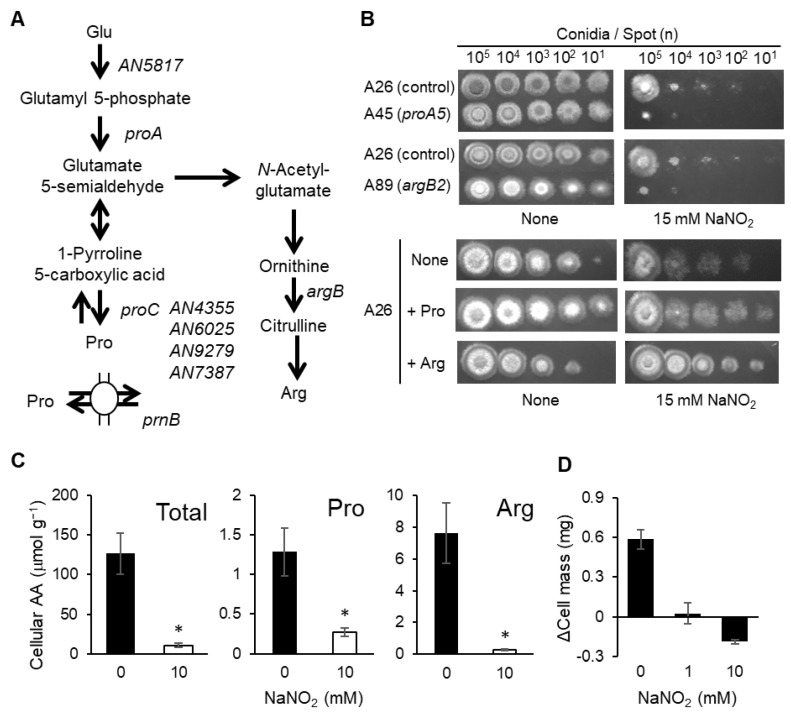
Nitric oxide-induced amino acid starvation in *A. nidulans* cells. (**A**) Biosynthetic pathways of proline and arginine in *A. nidulans*. (**B**) Morphology of colonies after incubating conidia (1 × 10^1^–10^5^) at 37 °C for 48 h on MMN agar without or with indicated amounts of NaNO_2_ (pH 5.5). Upper panel: A45 (*proA5*), A89 (*argB2*), and A26 (control; *pro*^+^, *arg*^+^) strains. Proline and arginine (1 mM each) were added to support auxotrophic growth, and 15 mM NaNO_2_ (pH 5.5) was also added. Bottom panel: A26 strain cultured on MMN with exogenous proline, arginine (10 mM each), and 15 mM NaNO_2_ (pH 5.5). (**C**) Cellular amino acid contents in liquid *A. nidulans* YMS9 cultures. Data are shown as means ± SD of three biological replicates (* *p* < 0.05, vs. 0 mM NaNO_2_; Student *t*-tests). (**D**) YMS9 cells were incubated for 18 h, followed by NaNO_2_ for 3 h. Then, changes in wet cell mass were evaluated.

This gene encoded a predicted 1-pyrroline 5-carboxylate reductase that might be involved in proline biosynthesis (Figure 2A). This enzyme was one of four (AN4355, AN6025, AN9279, and AN7387) encoded by the *A. nidulans* genome, with amino acid identities of 30–33%. None of the transformants in which the respective genes required proline for growth were susceptible to RNS (Supplementary Figure S1), suggesting that their encoded enzymes had redundant functions in proline biosynthesis. Although we did not further analyze the functions of these isozymes, the link between amino acid biosynthesis and NO tolerance indicated an unknown metabolic adaptation mechanism against RNS stress in this fungus.

We examined the roles of proline and arginine in RNS tolerance since their biosynthetic pathways share intermediates and are closely related (Figure 2A). Adding proline or arginine to MMN facilitated the growth of mutants with *proA5* and *argB2* alleles that cannot synthesize proline and arginine and thus conferred minimal protection against RNS stress (Figure 2B). The growth of the parental strain under RNS stress was increased by adding proline and arginine to the culture medium (Figure 2B). Acidified nitrite decreased the intracellular contents of proline and arginine (Figure 2C), some other amino acids (Supplementary Figure S2), and the total amino acids (Figure 2C). Incubation with RNS dose-dependently decreased the cell mass (Figure 2D). These results indicate that RNS attenuate cell growth by starving *A. nidulans* of at least the amino acids that are required for normal growth.

### 3.2. Cross-Pathway Control Mechanism Confers RNS Tolerance

**Figure 3:**
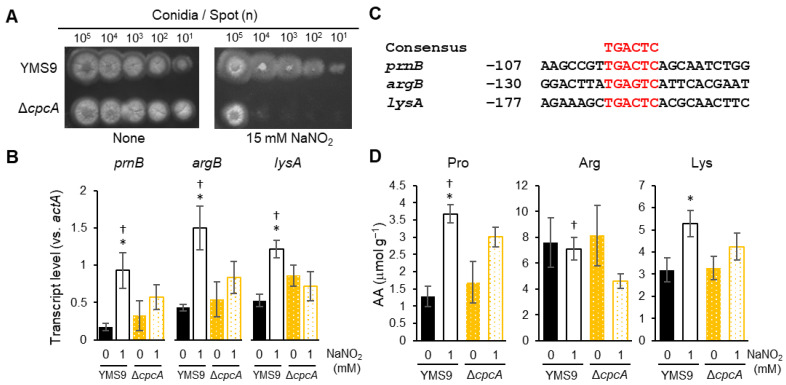
CpcA controls the gene expression of Pro and Arg-biosynthetic genes. (**A**) Conidia (1 × 10^1^–10^5^) were incubated at 37 °C for 48 h on MMN agar without or with 15 mM NaNO_2_ (pH 5.5), then colony morphology was assessed. (**B**) Relative ratios of parental strain (YMS9) and Δ*cpcA* transcripts to *actA*. Strains were incubated in MMN at 37 °C for 18 h and then without or with 10 mM NaNO_2_ (pH 5.5) for 3 h. Data are shown as means ± SD of data from three biological replicates (* *p* < 0.05 vs. YMS9 0 mM NaNO_2_; † *p* < 0.05 vs. Δ*cpcA* 1 mM NaNO_2_; Student *t*-tests). (**C**) Predicted CpcA-binding sequences in gene promoters. Nucleotides were predicted as numbers with translation start residues designated as 1. (**D**) Cellular amino acid content in liquid cultures of the parental strain (YMS9) and Δ*cpcA* incubated, as shown in (**C**). Data are shown as means ± SD of three biological replicates. (* *p* < 0.05, vs. YMS9 0 mM NaNO_2_; † *p* < 0.05, vs. Δ*cpcA* 1 mM NaNO_2_; Student *t*-tests). Δ*cpcA*, gene disruptant of *cpcA*.

Cross-pathway control is a fungal mechanism that responds to amino acid starvation, upregulates genes involved in amino acid biosynthesis, and controls general amino acid levels in the yeast *S. cerevisiae* [15,16,17]. We disrupted the fungal *cpcA* gene (Supplementary Figure S3) that encodes the master transcription regulator controlling this mechanism [17] and analyzed its function in the RNS response. The gene disruptant and the parental strain similarly thrived on MMN agar without affecting the morphology and conidia development. However, the growth of the disruptant was repressed compared with the parental strain on MMN agar containing 10 mM acidified nitrite (Figure 3A). We used a liquid medium containing 1 mM of acidified nitrite instead of 10 mM acidified nitrite in subsequent experiments to avoid damaging the cells (Figure 2D) and disrupting the mechanism of the CpcA-mediated RNS response. The qPCR findings showed that 1 mM acidified nitrite solution increased *cpcA* expression 1.4 ± 0.3 fold (*p* < 0.02) and indicated the importance of the cross-pathway control mechanism for growth under RNS stress. 

The present study found that RNS stress induced the expression of the *prnB* (AN1732), *argB* (AN4409), and *lysA* (AN2873) genes (Figure 2A and Figure 3B), which are controlled via CpcA in *A. nidulans* [19,28,29]. The *cpcA* gene disruptant did not significantly induce the expression of these genes. This indicated that CpcA mediates the response to RNS induction, possibly via CpcA binding to its consensus TGACTC sequence [29] in its gene promoter (Figure 3C). These results indicate that the fungus responds to RNS and induces *prnB*, *argB,* and *lysA* through CpcA-mediated cross-pathway control. We evaluated the intracellular levels of proline, arginine, and lysine (Figure 3D). The results indicate that RNS (1 mM acidic nitrite) increase the levels of proline and lysine along with the transcriptional induction of *prnB* and *lysA*. The levels of proline and arginine partly depended on intact *cpcA* under RNS stress, indicating that CpcA controls a mechanism that maintains these amino acids. These and other amino acids in the cells were quantified to understand the global regulation of amino acids (Supplementary Figure S4). The results suggest that *cpcA* significantly decreased (*p* < 0.05) proline, arginine, serine, cysteine, glycine, histidine, valine, leucine, isoleucine, and tryptophan levels in the null mutant of *cpcA*. Such an increase was not evident under normal conditions, revealing a hitherto unknown role of CpcA under RNS stress. We also found that RNS increased the levels of cellular proline, lysine, tyrosine, phenylalanine, and glutamine, whereas alanine, asparagine, threonine, methionine, glutamic, and aspartic acids did not significantly respond to RNS and were not *cpcA*-dependent. The content of these amino acids might have been underestimated due to intracellular consumption because numerous synthesizing genes were RNS and *cpcA* dependently upregulated (Figure 3 and transcriptome shown below). 

### 3.3. Transcription Responses of Amino Acid Biosynthesis to RNS

**Figure 4:**
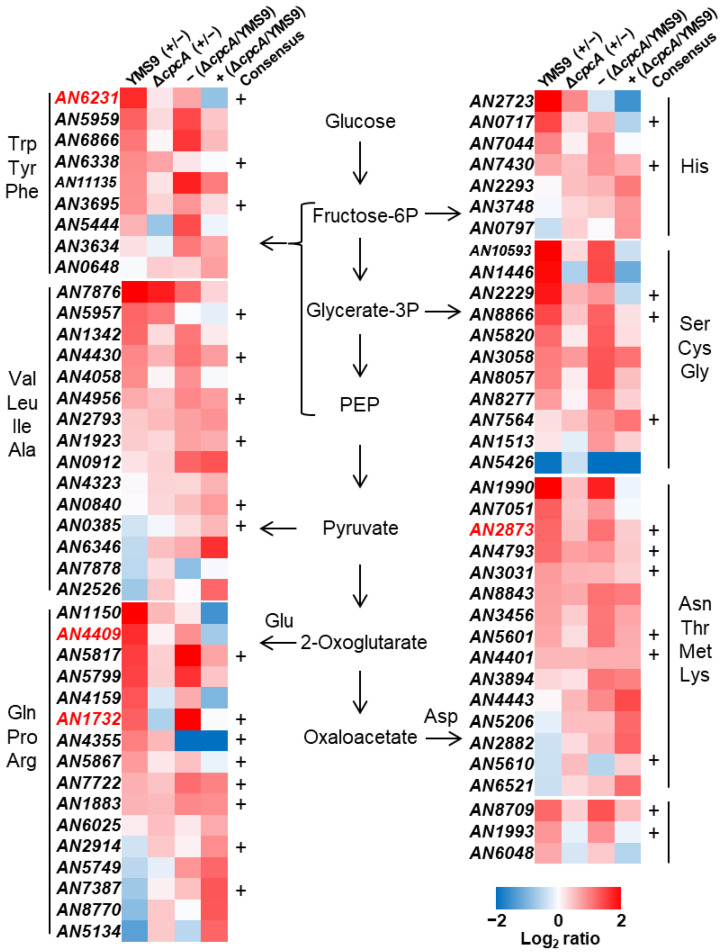
Transcriptomes of genes involved in amino acid synthesis. Parental (YMS9) and *cpcA* disruptant (Δ*cpcA*) strains were incubated in MMN medium at 37 °C for 18 h and then with (+) or without (−) 1 mM NaNO_2_ (pH 5.5) for 3 h. Genes were grouped based on the synthesized amino acids. +, CpcA-binding consensus on gene promoters. Red, *cpcA*-dependent genes.

The analysis of fungal transcriptomes affected by exposure to acidified nitrite and/or intact CpcA via mRNA sequencing identified 9,208 genes that were significantly expressed (*p* < 0.05), which accounted for 86% of the entire *A. nidulans* genome. We selected 76 genes encoding enzymes involved in amino acid synthesis (Supplementary Table S1). Figure 4 shows that incubating the parental strain with 1 mM NaNO_2_ at pH 5.5 increased the expression of 56 of these genes, among which 27 were increased > 2-fold (*p* < 0.01). These genes were distributed in the biosynthetic pathways of 20 amino acids. The number of genes regulated via RNS stress was notable. Disrupting *cpcA* alleviated the RNS-dependent increase in the transcripts of 22 of the 27 genes, indicating that CpcA is involved in inducing their RNS-dependent transcription. We analyzed amino acid synthesis gene promoters using Multiple EM for Motif Elicitation (MEME) v.5.5.1 and found the predicted binding sequence, 5′-TGACTC-3′, of CpcA [29] (Figure 4, “+”). By focusing on the amino acids that require CpcA for maintenance (Figure 3, Supplementary Figure S4), we identified genes encoding glutamate 5-kinase (AN5817), acetolactate synthase (AN4430 and AN4956), and 3-phosphoglycerate dehydrogenase (AN8866) that are, respectively, involved in the de novo synthesis of proline/arginine, valine/leucine/isoleucine, and serine/cysteine. These enzymes catalyze the initial step of synthesizing these respective amino acids and are consistent with the importance of CpcA in regulating the subsequent biosynthetic reactions of these amino acids. The transcription levels were decreased in Δ*cpcA* relative to the parental strain when incubated with RNS stress (Figure 4, two right columns). These results show that CpcA in cells starved of amino acids increases their synthesis and supports cell growth under RNS stress. This is a novel example of amino acid biosynthesis regulated via RNS stress in *A. nidulans*.

### 3.4. Global Transcription Was Altered by RNS and under Cross-Pathway Control

The fungal transcriptome was analyzed to determine the global transcription responses to RNS stress and amino acid starvation. We found that 266 genes that were upregulated (log_2_ ratio > 3.0) due to RNS stress were categorized into the gene ontology (GO) domain of the biological process (Table 2). These results indicate that the genes involved in RNS metabolic processes (GO:2001057, 0046209) and xenobiotic responses (GO:0006855, 0055085, and 0009410) were upregulated under RNS stress. This implied that fungal responses to RNS and toxic compounds are generated via RNS reactions with the cellular compounds that result in tolerance. The upregulated genes that encoded the proteins involved in the metabolic processes of nitrate and nitrite (GO:0042128, 0042126, 0015706, and 0015707), supported the notion that the fungus metabolizes some acidified nitrite, and presumably nitrate generated from RNS. 

Table 3 summarizes the ontology of genes with CpcA-dependent expression. Common GO terms between with and without RNS stress included secondary metabolic processes (GO: 0044550, 0019748). We found that CpcA was associated with polyketide metabolism (GO: 0030638, 0030639) in cells without RNS stress, and with mycotoxin, gliotoxin, and asperfuranone metabolism (GO: 0043385, 0043386, 2001308, 1900552, and 1900554) in cells with RNS stress. These results showed that CpcA upregulates the secondary metabolism of various compounds. Under RNS stress, CpcA is involved in the regulation of membrane transporters associated with mass intracellular and extracellular transport (GO: 0055085). Under normal conditions (without RNS stress), CpcA alters the transcriptome of intracellular respiration and energy metabolism (GO: 0019646, 0009060, and 0045333), including oxidative phosphorylation (GO: 0006119, 0042773, and 0042775). Among 266 RNS-inducible genes, the expression levels of 230 of them were CpcA-dependent (log_2_ ratio < 1.2 for RNS induction in the Δ*cpcA* strain), indicating a close correlation between RNS and CpcA-dependent gene expression.

### 3.5. Levels of Carbon Metabolism and Amino Acid Precursors Were Decreased Due to RNS

**Figure 5:**
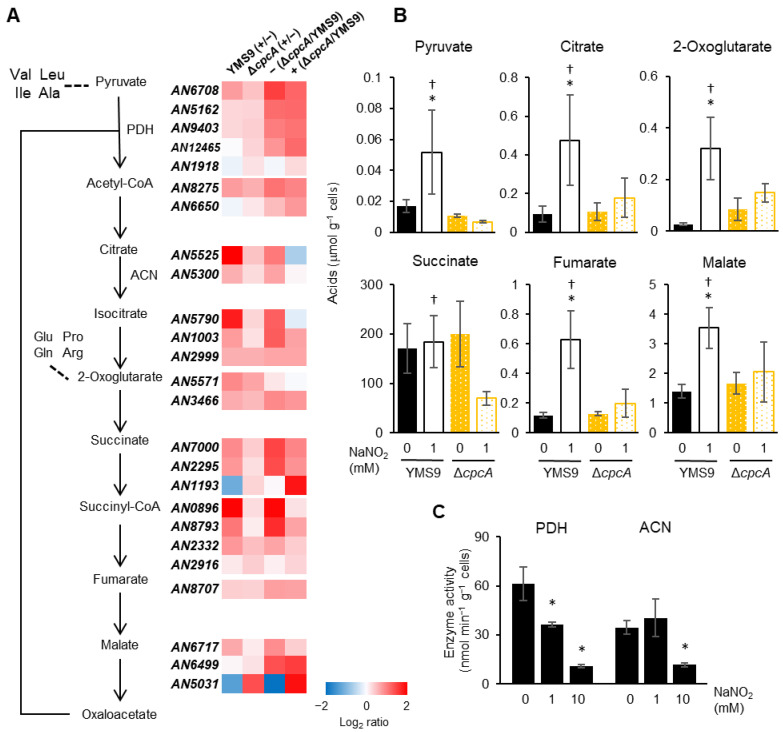
Intracellular organic acid synthesis is regulated via CpcA. (**A**) Gene expression of pyruvate and TCA cycle metabolism. The reanalysis of the transcriptome shown in Figure 4. (**B**) Organic acids in liquid cultures of *A. nidulans* strains (as shown in Figure 4). Data are means ± SD of data from three biological replicates (* *p* < 0.05, vs. YMS9 0 mM NaNO_2_; ^†^ *p* < 0.05, vs. Δ*cpcA* 1 mM NaNO_2_; Student *t*-tests). (**C**) Activity of PDH and ACN in fungal cell-free extracts. Data are shown as means ± SD of three biological replicates (* *p* < 0.01, vs. 0 mM NaNO_2_; Student *t*-tests). ACN, aconitase; NaNO_2_, sodium nitrite PDH, pyruvate dehydrogenase.

We analyzed the expression of genes in the transcriptome that were involved in pyruvate and TCA cycle metabolism. Moderate levels of RNS increased the expression of the genes encoding the E1 and E2 components of pyruvate dehydrogenase (AN6708, AN5162, and AN9403) and 15 of 20 genes encoding TCA cycle enzymes (Figure 5A; Supplementary Table S1 for annotations). The intracellular levels of pyruvate, citrate, 2-oxoglutarate, fumarate, and malate were consistently increased under RNS stress (Figure 5B), indicating that *A. nidulans* activates the anabolic mechanisms of these OAs in response to RNS. Notably, none of these were increased in the gene disruptant of *cpcA* under RNS stress (Figure 5B). The transcriptome indicated that the RNS-dependent induction of some, but not all, genes in the TCA cycle was decreased in the fungal strain harboring a *cpcA* null mutation compared with the parental strain and included genes for aconitase, isocitrate dehydrogenase, succinyl-CoA synthase, succinate dehydrogenase, and malate dehydrogenase (Figure 5A). These results indicated that CpcA regulated the synthesis of these OAs along with global amino acids under RNS stress. 

Fungal pyruvate dehydrogenase and aconitase are RNS-sensitive thiolate enzymes [9]. High levels of RNS inhibited pyruvate dehydrogenase and aconitase activities in *A. nidulans* cells (Figure 5C). A limited supply of biosynthetic amino acid precursors could trigger an intracellular deficiency of amino acids and explain the activation of the cross-pathway control mechanism. 

## 4. Discussion

**Figure 6:**
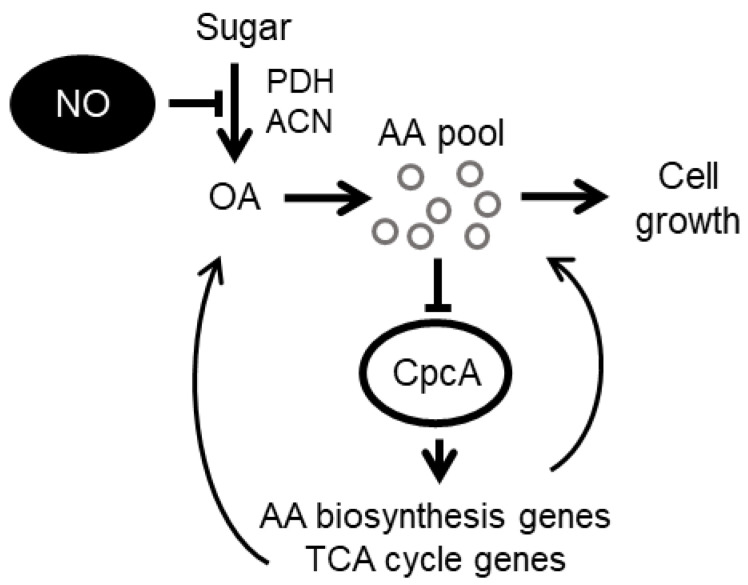
Metabolic regulation mechanism counteracts RNS for fungal growth. AA, amino acid; CpcA, C-phycocyanin alpha subunit; NO, nitric oxide; OA, organic acid; RNS, reactive nitrogen species; TCA, tricarboxylic acid.

This study revealed that RNS induces intracellular amino acid starvation, and in response, a cross-pathway control mechanism activates the biosynthesis of amino acids to maintain appropriate levels, enable RNS tolerance, and support growth. This can be explained as follows (Figure 6). The powerful oxidative capacity of RNS inhibits the activity of thiolate enzymes, such as pyruvate dehydrogenase and aconitase, which are involved in carbon metabolism [9] (Figure 5C). This reduces the levels of intracellular amino acid precursors and the intracellular amino acid pool. Consequential amino acid starvation elicits a CpcA response that activates global amino acid biosynthesis at the transcriptional level to maintain the amino acid pool and, consequently, cell growth. The ability of CpcA to compensate for a defective amino acid pool and maintain cell growth becomes insufficient under high RNS stress. This novel fungal mechanism of adaptation to RNS stress regulates intracellular metabolism and is distinct from the established detoxification mechanisms.

Although RNS inhibit amino acid biosynthetic mechanisms in bacteria, this has never been identified in fungi until now. Incubating *E. coli* with NO inactivates the iron-sulfur enzyme dihydroxy acid dehydratase, which is involved in the synthesis of branched-chain amino acids and leads to their depletion [30,31]. Nitric oxide inactivates the iron-sulfur enzyme aconitase and other TCA cycle enzymes that synthesize the precursors of methionine and lysine in *Salmonella typhimurium*, and results in auxotrophy [32]. Taken together, the presented findings suggest that repressing amino acid biosynthesis by inhibiting RNS-sensitive enzymes is widespread in microorganisms. In contrast, the inhibited biosynthesis of amino acid species that are required for growth under RNS differs among species, possibly as a result of variable sensitivity to metabolic enzymes or the amounts of amino acid required to support normal growth. 

The apparent primary function of cross-pathway control is to enhance amino acid biosynthesis in response to amino acid starvation. Our transcriptome and biochemical findings revealed that an *A. nidulans* cross-pathway control mechanism activates pyruvate and the metabolism of other OAs at the transcriptional level. General amino acid control in yeast involves the biosynthesis of organic and amino acids. During this process, Gcn4p activates the expression of the lipoamide dehydrogenase (*LPD1*) gene that subsequently increases the activities of PDH and 2-oxoglutarate dehydrogenase. These enzymes then form complexes with LPD1 [33], which supplies cells with amino acid precursors. In contrast, the expression of the LPD1 ortholog of *A. nidulans* (see transcriptome data) is not deregulated in the absence of CpcA, but rather, CpcA activates PDH and 2-oxoglutarate dehydrogenase gene expression (Figure 5A). Thus, fungal cross-pathway control seems to regulate the expression of many metabolic genes. 

We considered that the versatile chemotoxic effects of NO and RNS on cells regulate global gene expression. This is consistent with the finding that RNS stress alters the transcription of the *A. nidulans* genes involved in intracellular metabolic activities, such as the TCA cycle, amino acid synthesis, and oxidative stress responses (Table 2). The responses of fungal transcription factors to RNS remain unclear. A NO donor regulates the transcription of numerous genes in some yeasts as well as in *A. nidulans* [34,35,36]. *S. cerevisiae* Fzf1p responds to RNS and activates the transcription of the *FHB1* gene encoding flavohemoglobin [37], but the mechanism through which RNS are directly sensed remains unknown. We propose a novel RNS-sensing mechanism mediated via RNS-induced changes in intracellular metabolites (amino acids). Exploring not only RNS sensors but also the response mechanisms to various metabolites might provide insight into fungal RNS tolerance.

## Figures and Tables

**Table 1:** Aspergillus nidulans strains.

Strain	Genotype	Source
YMS9	*yA2*; *pyrG89*; *pyroA4* (progeny of a meiotic cross between ABPU1 and A952)	Reference [9]
pRG3	*yA2*; *pyrG89*; *pyroA4*; pRG3-AMA1	This study
pRG3-1.7	*yA2*; *pyrG89*; *pyroA4*; pRG3-AMA1.7	This study
pRG3-4354	*yA2*; *pyrG89*; *pyroA4*; pRG3-AMA1.7_*AN4354*	This study
pRG3-4355	*yA2*; *pyrG89*; *pyroA4*; pRG3-AMA1.7_*AN4355*	This study
TN02A3	*pyrG89*; *argB2*; *nkuA::argB*; *pyroA4*	FGSC
Δ*AN4355*	*pyrG89*; *argB2*; *nkuA::argB*; *pyroA4*; Δ*AN4355*::*pyrG*	This study
Δ*AN6025*	*pyrG89*; *argB2*; *nkuA::argB*; *pyroA4*; Δ*AN6025*::*pyrG*	This study
Δ*AN7387*	*pyrG89*; *argB2*; *nkuA::argB*; *pyroA4*; Δ*AN7387*::*pyrG*	This study
Δ*AN9279*	*pyrG89*; *argB2*; *nkuA::argB*; *pyroA4*; Δ*AN9279*::*pyrG*	This study
A26	*biA1*	FGSC
A45	*biA1*; *proA5*	FGSC
A89	*biA1*; *argB2*	FGSC
Δ*cpcA*	*yA2*; *pyrG89*; *pyroA4*; Δ*cpcA*::*pyrG*	This study

FGSC, Fungal Genetic Stock Center, University of Kansas Medical Center, Kansas City, KS, USA).

**Table 2:** Gene ontology terms enriched among the genes regulated via RNS stress.

GO ID	GO Term	n	*p*
With and without NO_2_^−^ (pH 5.5); YMS9, log_2_ > 3			
GO:0055085	Transmembrane transport	33	0.001
GO:0044282	Small-molecule catabolic process	11	0.04
GO:0006855	Xenobiotic transmembrane transport	6	<0.001
GO:0042908	Xenobiotic transport	6	0.002
GO:2001057	Reactive nitrogen species metabolic process	5	<0.001
GO:0043648	Dicarboxylic acid metabolic process	5	0.03
GO:1901606	Alpha-amino acid catabolic process	5	0.04
GO:0042128	Nitrate assimilation	4	<0.001
GO:0042126	Nitrate metabolic process	4	<0.001
GO:0071941	Nitrogen cycle metabolic process	4	<0.001
GO:0009410	Response to xenobiotic stimuli	3	0.007
GO:0006536	Glutamate metabolic process	3	0.01
GO:0009065	Glutamine family amino acid catabolic process	3	0.01
GO:0006083	Acetate metabolic process	3	0.02
GO:0015074	DNA integration	3	0.02
GO:0015706	Nitrate transport	2	<0.001
GO:0046209	Nitric oxide metabolic process	2	0.002
GO:0015707	Nitrite transport	2	0.002
GO:0045807	Positive regulation of endocytosis	2	0.009
GO:0045041	Protein import into mitochondrial intermembrane spaces	2	0.02
GO:0071466	Cellular response to xenobiotic stimulus	2	0.02
GO:0015740	C4-dicarboxylate transport	2	0.02
GO:0032196	Transposition	2	0.02
GO:0033609	Oxalate metabolic process	2	0.02
GO:0006538	Glutamate catabolic process	2	0.02
GO:0043649	Dicarboxylic acid catabolic process	2	0.03
GO:0030100	Regulation of endocytosis	2	0.03
GO:0043942	Negative regulation of sexual sporulation resulting in the formation of a cellular spore	2	0.05

**Table 3:** Gene ontology terms enriched among the genes regulated via CpcA.

GO ID	GO Term	n	*p*
YMS9 vs. Δ*cpcA* without NO_2_^−^ (pH 5.5); log_2_ > 2			
GO:0009058	Biosynthetic process	21	0.040
GO:0044550	Secondary metabolite biosynthetic process	17	<0.001
GO:0019748	Secondary metabolic process	17	<0.001
GO:0030639	Polyketide biosynthetic process	4	<0.001
GO:0030638	Polyketide metabolic process	4	<0.001
GO:0006091	Generation of precursor metabolites and energy	4	0.044
GO:0019646	Aerobic electron transport chain	3	0.002
GO:0042775	Mitochondrial ATP-synthesis-coupled electron transport	3	0.002
GO:0042773	ATP-synthesis-coupled electron transport	3	0.002
GO:0006119	Oxidative phosphorylation	3	0.002
GO:0022904	Respiratory electron transport chain	3	0.003
GO:0022900	Electron transport chain	3	0.004
GO:1901606	Alpha-amino acid catabolic process	3	0.01
GO:0009060	Aerobic respiration	3	0.02
GO:0009063	Cellular amino acid catabolic process	3	0.02
GO:0045333	Cellular respiration	3	0.02
GO:0046034	ATP metabolic process	3	0.02
GO:0015980	Energy derivation via the oxidation of organic compounds	3	0.04
GO:0006123	Mitochondrial electron transport, cytochrome c to oxygen	2	0.001
GO:0009081	Branched-chain amino acid metabolic process	2	0.02
YMS9 vs. Δ*cpcA* with NO_2_^−^ (pH 5.5); log_2_ > 2			
GO:0055114	Obsolete oxidation-reduction process	52	<0.001
GO:0055085	Transmembrane transport	47	<0.001
GO:0019748	Secondary metabolic process	35	0.002
GO:0044550	Secondary metabolite biosynthetic process	33	0.003
GO:0042908	Xenobiotic transport	11	<0.001
GO:0006855	Xenobiotic transmembrane transport	9	<0.001
GO:0008645	Hexose transmembrane transport	6	0.008
GO:0015749	Monosaccharide transmembrane transport	6	0.008
GO:0034219	Carbohydrate transmembrane transport	6	0.009
GO:0008643	Carbohydrate transport	6	0.02
GO:0046323	Glucose import	5	0.02
GO:1904659	Glucose transmembrane transport	5	0.02
GO:0009410	Response to xenobiotic stimuli	4	0.001
GO:0015698	Inorganic anion transport	4	0.02
GO:0009636	Response to toxic substances	4	0.04
GO:0006577	Amino acid betaine metabolic process	3	0.004
GO:0043386	Mycotoxin biosynthetic process	3	0.02
GO:0043385	Mycotoxin metabolic process	3	0.03
GO:0006578	Amino acid betaine biosynthetic process	2	0.02
GO:0006528	Asparagine metabolic process	2	0.03
GO:0033609	Oxalate metabolic process	2	0.03
GO:0071466	Cellular response to xenobiotic stimuli	2	0.03
GO:2001308	Gliotoxin metabolic process	2	0.04
GO:0015802	Basic amino acid transport	2	0.04
GO:2001310	Gliotoxin biosynthetic process	2	0.04
GO:0006817	Phosphate ion transport	2	0.04
GO:1900554	Asperfuranone biosynthetic process	2	0.04
GO:1902644	Tertiary alcohol metabolic process	2	0.04
GO:1902645	Tertiary alcohol biosynthetic process	2	0.04
GO:1900552	Asperfuranone metabolic process	2	0.04
GO:0042727	Favin-containing compound biosynthetic process	2	0.04

## Data Availability

The data supporting the conclusions of this study are available and included within the article.

## Supplementary Materials

The following supporting information can be downloaded at https://www.mdpi.com/article/10.3390/jof10010058/s1. Table S1. Genes for synthesizing amino acids and the TCA cycle. Table S2. Forward and reverse primer sequences. Figure S1. Construction of *proC* paralog gene disruptants. Figure S2. Acidified nitrite (10 mM) induces general amino acid starvation in fungal cells. Figure S3. Construction of *cpcA* gene disruptant. Figure S4. Low concentration of acidified nitrite induced intracellular starvation of general amino acids.
