# Supplementary material for: The Metabolic Regulation of Amino Acid Synthesis Counteracts Reactive Nitrogen Stress via Aspergillus nidulans Cross-Pathway Control
Source: J Fungi (Basel). 2024 Jan 10;10(1):58. doi: 10.3390/jof10010058 (PMC10817288; doi:10.3390/jof10010058)
Supplement: Supplementary file 1 [file jof-10-00058-s001.zip › jof-2797804-supplementary.pdf]

Supplementary materials

The Metabolic Regulation of Amino Acid Synthesis Counteracts Reactive Nitrogen Stress via *Aspergillus nidulans* Cross-Pathway Control

Madoka Amahisa [1], Madoka Tsukagoshi [1], Chihiro Kadooka [1], Shunsuke Masuo [1], Norio Takeshita [1], Yuki Doi [1], Hiroshi Takagi [2] and Naoki Takaya [1,*]

[1] Microbiology Research Center for Sustainability, Faculty of Life and Environmental Sciences, University of Tsukuba, 1-1-1 Tennodai, Tsukuba 305-8572, Japan
[2] Institute for Research Initiatives, Nara Institute of Science and Technology, 8916-5 Takayama, Ikoma 630-0192, Japan
* Correspondence: takaya.naoki.ge@u.tsukuba.ac.jp

Supplementary Table S1. Genes for synthesizing amino acids and TCA cycle.

Gene ID	Function
Amino acid biosynthesis	
AN0717	histidinol-phosphate aminotransferase [EC:2.6.1.9]
AN0797	phosphoribosyl-ATP pyrophosphohydrolase / phosphoribosyl-AMP cyclohydrolase / histidinol dehydrogenase [EC:3.6.1.31 3.5.4.19 1.1.1.23]
AN2293	phosphoribosylformimino-5-aminoimidazole carboxamide ribotide isomerase [EC:5.3.1.16]
AN2723	histidinol dehydrogenase [EC:1.1.1.23]
AN3748	ATP phosphoribosyltransferase [EC:2.4.2.17]
AN7044	histidinol-phosphatase (PHP family) [EC:3.1.3.15]
AN7430	imidazole glycerol-phosphate synthase [EC:4.3.2.10]
AN0648	hypothetical protein with a predicted role in tryptophan biosynthesis
AN3634	anthranilate phosphoribosyltransferase [EC:2.4.2.18]
AN3695	anthranilate synthase component I [EC:4.1.3.27]
AN5444	tryptophan synthase [EC:4.2.1.20]
AN5959	prephenate dehydrogenase ($NADP^+$) [EC:1.3.1.13]
AN6231	tryptophan synthase [EC:4.2.1.20]
AN6338	aromatic amino acid aminotransferase I / 2-aminoadipate transaminase [EC:2.6.1.57 2.6.1.39 2.6.1.27 2.6.1.5]
AN6866	chorismate mutase [EC:5.4.99.5]
AN11135	Prephenate dehydratase involved in phenylalanine biosynthesis
AN1446	cystathionine gamma-lyase [EC:4.4.1.1]
AN1513	cysteine synthase [EC:2.5.1.47]
AN2229	homoserine *O*-acetyltransferase/*O*-succinyltransferase [EC:2.3.1.31 2.3.1.46]
AN3058	glycine hydroxymethyltransferase [EC:2.1.2.1]
AN5426	threonine aldolase [EC:4.1.2.48]
AN5820	cystathionine beta-synthase [EC:4.2.1.22]
AN7564	threonine aldolase [EC:4.1.2.48]
AN8057	cysteine synthase [EC:2.5.1.47]
AN8277	*O*-acetylhomoserine/*O*-acetylserine sulfhydrylase [EC:2.5.1.49 2.5.1.47]
AN8866	D-3-phosphoglycerate dehydrogenase / 2-oxoglutarate reductase [EC:1.1.1.95 1.1.1.399]
AN10593	phosphoserine phosphatase involved in L-serine biosynthesis
AN0385	branched-chain amino acid aminotransferase [EC:2.6.1.42]
AN0840	2-isopropylmalate synthase [EC:2.3.3.13]
AN0912	3-isopropylmalate dehydrogenase [EC:1.1.1.85]
AN1342	alanine-glyoxylate transaminase / serine-glyoxylate transaminase / serine-pyruvate transaminase [EC:2.6.1.44 2.6.1.45 2.6.1.51]
AN1923	alanine transaminase [EC:2.6.1.2]
AN2526	ketol-acid reductoisomerase [EC:1.1.1.86]
AN2793	3-isopropylmalate dehydrogenase [EC:1.1.1.85]
AN4058	dihydroxy-acid dehydratase [EC:4.2.1.9]
AN4323	branched-chain amino acid aminotransferase [EC:2.6.1.42]
AN4430	acetolactate synthase I/III small subunit [EC:2.2.1.6]
AN4956	acetolactate synthase I/II/III large subunit [EC:2.2.1.6]
AN5957	branched-chain amino acid aminotransferase [EC:2.6.1.42]
AN6346	dihydroxy-acid dehydratase [EC:4.2.1.9]

AN7876	branched-chain amino acid aminotransferase [EC:2.6.1.42]
AN7878	branched-chain amino acid aminotransferase [EC:2.6.1.42]
AN1990	homocitrate synthase [EC:2.3.3.14]
AN2873	ubiquitin carboxyl-terminal hydrolase 16 [EC:3.4.19.12]
AN2882	homoserine dehydrogenase [EC:1.1.1.3]
AN3031	threonine synthase [EC:4.2.3.1]
AN3456	cystathionine gamma-synthase [EC:2.5.1.48]
AN3894	homoaconitase [EC:4.2.1.-]
AN4401	asparagine synthase (glutamine-hydrolyzing) [EC:6.3.5.4]
AN4443	5-methyltetrahydropteroyltriglutamate--homocysteine methyltransferase [EC:2.1.1.14]
AN4793	aspartate-semialdehyde dehydrogenase [EC:1.2.1.11]
AN5206	homoisocitrate dehydrogenase [EC:1.1.1.87]
AN5601	saccharopine dehydrogenase ($NADP^+$, L-glutamate forming) [EC:1.5.1.10]
AN5610	L-2-aminoadipate reductase [EC:1.2.1.95]
AN6521	homoaconitate hydratase [EC:4.2.1.36]
AN7051	cysteine-*S*-conjugate beta-lyase [EC:4.4.1.13]
AN8843	homoserine kinase [EC:2.7.1.39]
AN1150	acetylornithine aminotransferase [EC:2.6.1.11]
AN1732	Proline-specific permease
AN1883	argininosuccinate synthase [EC:6.3.4.5]
AN2914	argininosuccinate lyase [EC:4.3.2.1]
AN4159	glutamine synthetase [EC:6.3.1.2]
AN4355	pyrroline-5-carboxylate reductase [EC:1.5.1.2]
AN4409	ornithine carbamoyltransferase [EC:2.1.3.3]
AN5134	glutamate synthase (NADH) [EC:1.4.1.14]
AN5749	acetylornithine deacetylase [EC:3.5.1.16]
AN5799	glutamate-5-semialdehyde dehydrogenase [EC:1.2.1.41]
AN5817	glutamate 5-kinase [EC:2.7.2.11]
AN5867	amino-acid *N*-acetyltransferase [EC:2.3.1.1]
AN6025	pyrroline-5-carboxylate reductase [EC:1.5.1.2]
AN7387	pyrroline-5-carboxylate reductase [EC:1.5.1.2]
AN7722	glutamate *N*-acetyltransferase / amino-acid *N*-acetyltransferase [EC:2.3.1.35 2.3.1.1]
AN8770	*N*-acetyl-gamma-glutamyl-phosphate reductase / acetylglutamate kinase [EC:1.2.1.38 2.7.2.8]
AN1993	aspartate aminotransferase, mitochondrial [EC:2.6.1.1]
AN6048	aspartate aminotransferase, cytoplasmic [EC:2.6.1.1]
AN8709	aspartate aminotransferase, cytoplasmic [EC:2.6.1.1]

TCA cycle and pyruvate dehydrogenase

AN5162	pyruvate dehydrogenase E1 component alpha subunit [EC:1.2.4.1]
AN9403	pyruvate dehydrogenase E1 component beta subunit [EC:1.2.4.1]
AN6708	pyruvate dehydrogenase E2 component (dihydrolipoamide acetyltransferase) [EC:2.3.1.12]
AN12465	Dihydrolipoamide dehydrogenase; the lipoamide dehydrogenase component (E3)
AN1918	phosphoenolpyruvate carboxykinase (ATP) [EC:4.1.1.49]
AN6650	citrate synthase [EC:2.3.3.1]
AN8275	citrate synthase [EC:2.3.3.1]
AN5300	aconitate hydratase [EC:4.2.1.3]
AN5525	aconitate hydratase [EC:4.2.1.3]
AN2999	isocitrate dehydrogenase [EC:1.1.1.42]

AN1003	isocitrate dehydrogenase (NAD^+) [EC:1.1.1.41]
AN5790	isocitrate dehydrogenase (NAD^+) [EC:1.1.1.41]
AN5571	2-oxoglutarate dehydrogenase E1 component [EC:1.2.4.2]
AN3466	2-oxoglutarate dehydrogenase E2 component (dihydrolipoamide succinyltransferase) [EC:2.3.1.61]
AN1193	succinyl-CoA synthetase alpha subunit [EC:6.2.1.4 6.2.1.5]
AN2295	succinyl-CoA synthetase alpha subunit [EC:6.2.1.4 6.2.1.5]
AN7000	succinyl-CoA synthetase beta subunit [EC:6.2.1.4 6.2.1.5]
AN8793	succinate dehydrogenase (ubiquinone) cytochrome b560 subunit
AN2916	succinate dehydrogenase (ubiquinone) flavoprotein subunit [EC:1.3.5.1]
AN2332	succinate dehydrogenase (ubiquinone) iron-sulfur subunit [EC:1.3.5.1]
AN0896	succinate dehydrogenase (ubiquinone) membrane anchor subunit
AN8707	fumarate hydratase, class II [EC:4.2.1.2]
AN6717	malate dehydrogenase [EC:1.1.1.37]
AN5031	malate dehydrogenase [EC:1.1.1.37]
AN6499	malate dehydrogenase [EC:1.1.1.37]

Supplementary Table S2. Forward (F) and reverse (R) primer sequences

Primers		Sequence (5’ → 3’)	Application
Screen			
oMN33_3	F	CTGTTCACTATGTCTTGAACTC	
oMN35_5	R	GTTGTAAAACGACGGCCAGTG	
AN4355	F	CGTCAGGGTACCGAGTTATAGAGCTCGGA	
AN4355+d500	R	CGTCAGGGTACCGATTACGGATGGCCCTTG	
AN4354	F	CGTCAGGGTACCTCCCTCGTGTTGCGGCC	
AN4354+d500	R	CGTCAGGGTACCGCGGATGCTCCTCTGTG	
Gene disruption			
cpcA_up	F	GGCCCTATGACTCACACCGG	5’-end of cpcA
cpcA_up	R	CTTTCGACAGGTATCGAATTCGTTGCGACGGTGATTTATC	5’-end of cpcA
cpcA_dw	F	TCTGGTAGACAAGCACTGAAAGAAGCTCTTCCGTTGTGGC	3’-end of cpcA
cpcA_dw	R	GCTAGGTAGGTATCGCAGTCG	3’-end of cpcA
cpcA_nested	F	GTGGACTAGGCGGGGAGAC	Nested primers
cpcA_nested	R	CCGTACTCCAAGAGGCGTGCT	Nested primers
pyrG	F	CTGACATCAGGCCAGAAC	pyrG
pyrG	R	CGCATATCTCTGGTTGGAG	pyrG
AN4355_up	F	CTTGGTCTTTCCACCTGTAC	5’-end of AN4355
AN4355_up	R	GCACTGGAATAGCTCCTAGGGATGAAGAGACTGGCGTATTC	5’-end of AN4355
AN4355_dw	F	CCTCAGGACAAGTCGACCTGGAGCATGGATATGACTGAGTC	3’-end of AN4355
AN4355_dw	R	CTCTGCTCCGAGAGTAATAGG	3’-end of AN4355
AN4355_nested	F	CGTACACAGTACGCGACTAC	Nested primers
AN4355_nested	R	GTGGGACTTATGCATGAGAC	Nested primers
AN6025_up	F	CAATACCTACCAGGCGTACCTG	5’-end of AN6025
AN6025_up	R	GCACTGGAATAGCTCCTAGGGATGGCCGAGATACACCTG	5’ end of AN6025
AN6025_dw	F	CCTCAGGACAAGTCGACCTGCAGCTTACAGGGAGTGGATG	3’ end of AN6025
AN6025_dw	R	GACGTCTGTCAAAGCCAGG	3’ end of AN6025
AN6025_nested	F	CGGCTTATAACGGGATTGC	Nested primers
AN6025_nested	R	CAATGTCCCAGCCAGTCTAC	Nested primers
AN7387_up	F	GTCAAAGACGCGATACGTTC	5’ end of AN7387
AN7387_up	R	GCACTGGAATAGCTCCTAGGGTCTTTGGAGACGAGTGGG	5’end of AN7387
AN7387_dw	F	CCTCAGGACAAGTCGACCTGCAACCGCCACCTCTATCTG	3’end of AN7387
AN7387_dw	R	CCATATAATGTCGTCGGGTG	3’-end of AN7387
AN7387_nested	F	GCCATGCGGATAAAGAGTC	Nested primers
AN7387_nested	R	GGCACCAGAATCTACGACC	Nested primers
AN9279_up	F	GGGCTGCACTATACAGGCTC	5’-end of AN9279
AN9279_up	R	GCACTGGAATAGCTCCTAGGGTGAATCGCTCGTAGGGTC	5’-end of AN9279
AN9279_dw	F	CCTCAGGACAAGTCGACCTGGTCTAGGCCAGAATGCATCAC	3’-end of AN9279
AN9279_dw	R	ATGCGTACGAGGCAGTCAC	3’-end of AN9279
AN9279_nested	F	CGCACATCTACAGGAATCTCC	Nested primers
AN9279_nested	R	CATCTTGTAAATCGTCATGCC	Nested primers
**Q-PCR**			
actA_RT	F	CAAGTCCAACCGTGAGAAGATG	actA
actA_RT	R	CGGAAGCATACAGGGAGAGAA	actA
prnB_RT	F	CGGAGGATGTATTGGCACTG	prnB
prnB_RT	R	TCATCAGCAAAGGAGCAGGA	prnB
argB_RT	F	CTTGTTCGCAATGCCTCCTC	argB
argB_RT	R	TTGTCCTCGTGCTTCGTTTG	argB
lysA_RT	F	AGCCTTATGCCAACCAAGACC	lysA
lysA_RT	R	GCACAGCACCCTTACCACAA	lysA

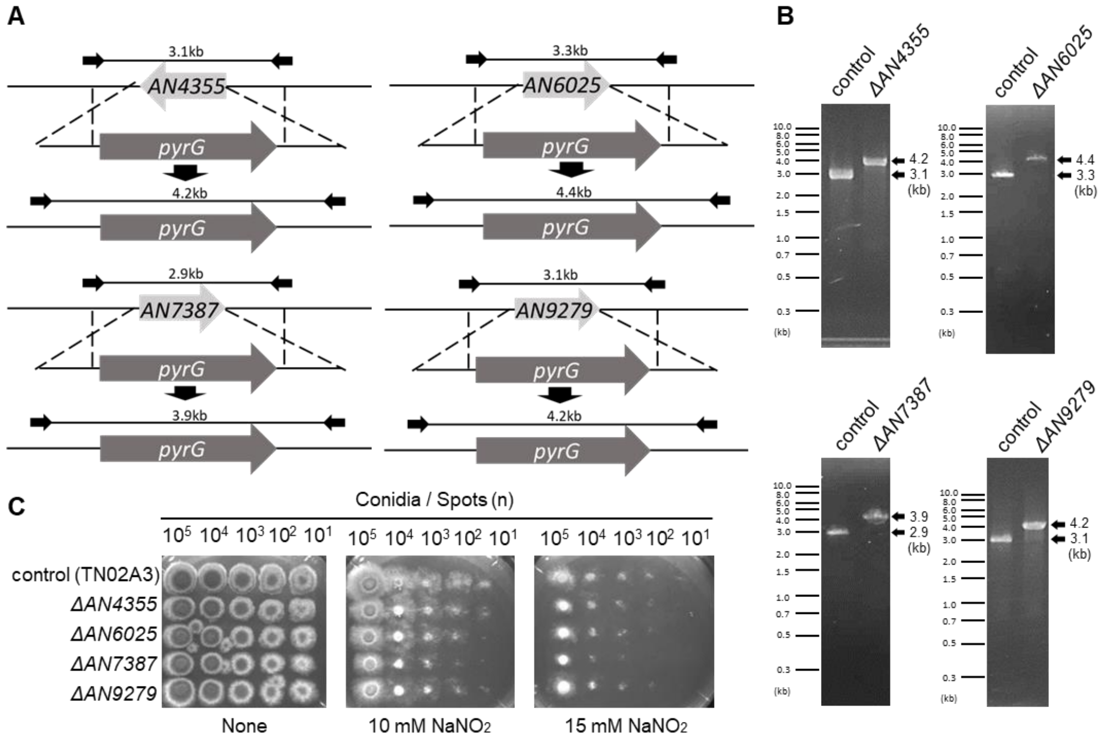

Supplementary Figure S1. Construction of *proC* paralog gene disruptants.

(A) Schematic representation of gene disruption. Arrows indicate positions and directions of primers (Table S1). (B) Disruption of transformant genes confirmed by PCR using primers and total DNA. (C) Morphology of gene disruptants and the parental TN02A3 strain.

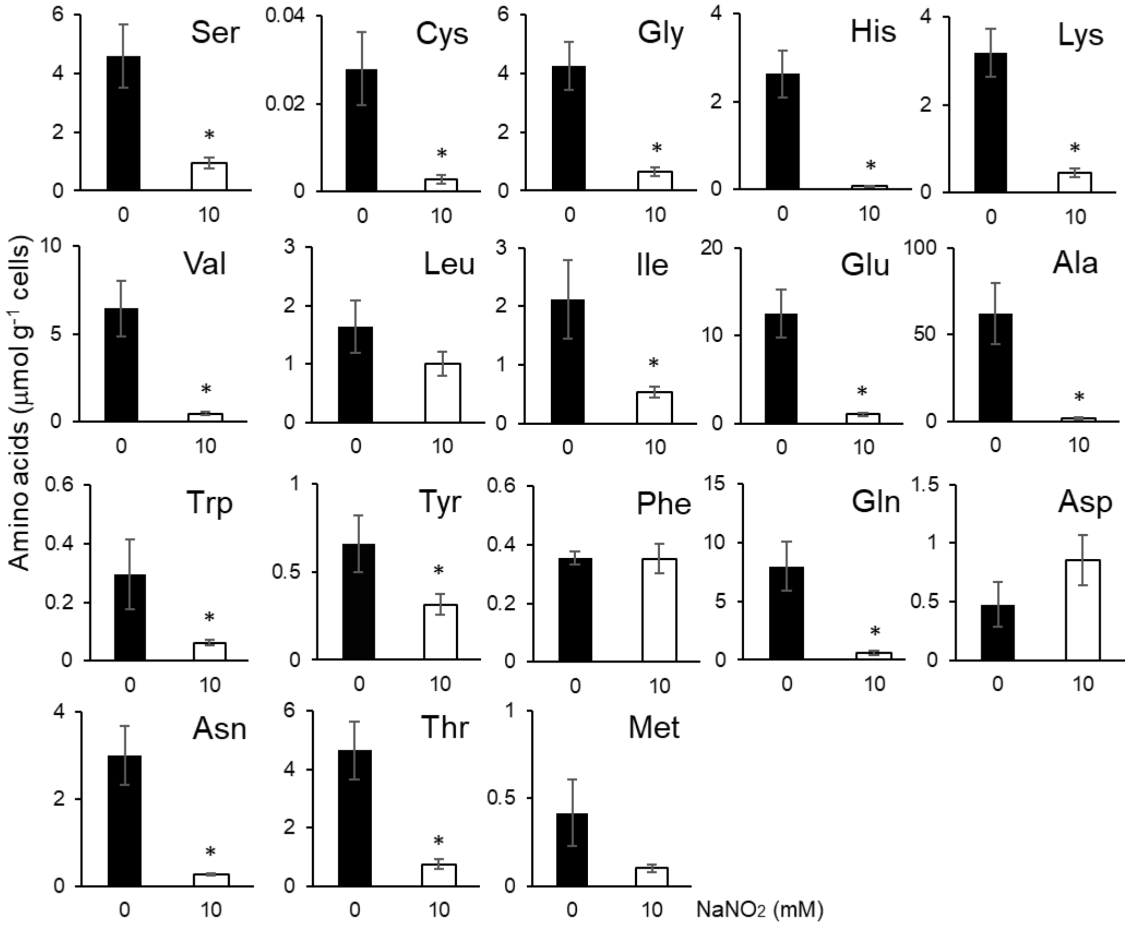

Supplementary Figure S2. Acidified nitrite (10 mM) induces general amino acid starvation in fungal cells.

Strain YMS9 was cultured in MMN medium at 37°C for 18 h, then with or without 10 mM $NaNO_2$ (pH 5.5) for 3 h. Cellular amino acid contents were determined LC-MS/MS as described in Materials and methods. Data are means ± SD of data from three biological replicates (**P* < 0.05, *vs* 0 mM $NaNO_2$ by Student's *t*-test).

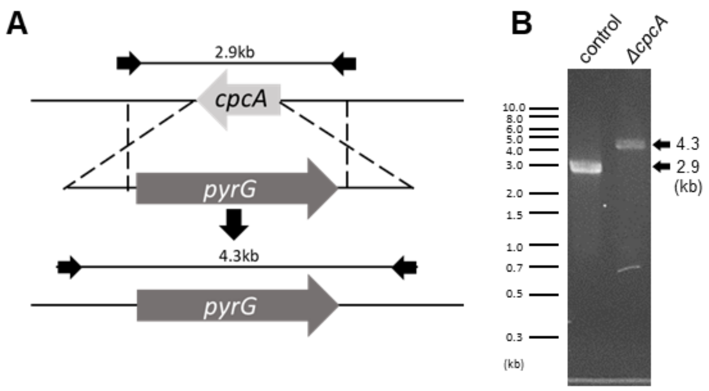

Supplementary Figure S3. Construction of *cpcA* gene disruptant.

(A) Schematic representation of gene disruption. Arrows indicate positions and directions of primers (Table S1). (B) PCR using primers and total DNA confirmed transformant gene disruption.

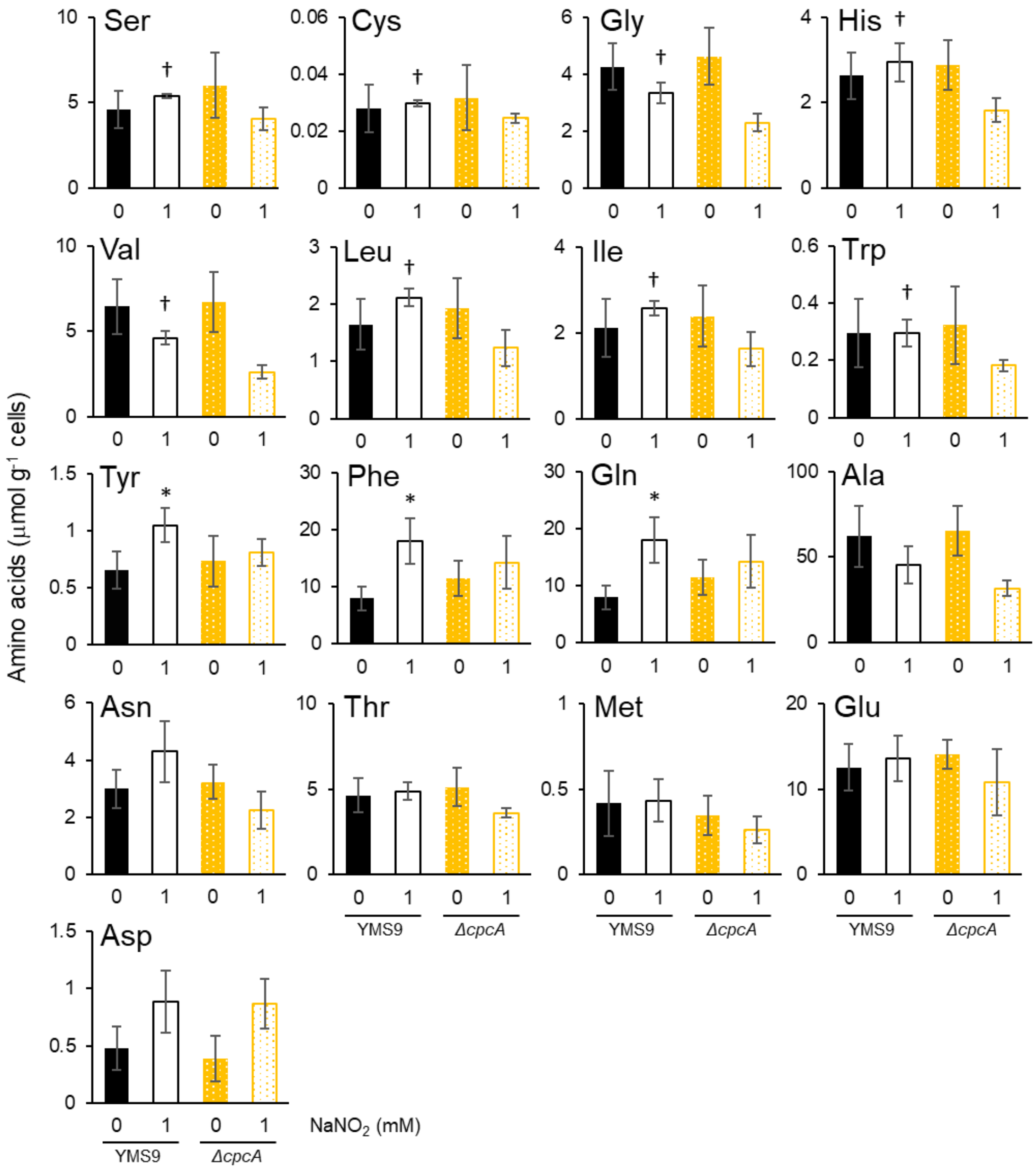

Supplementary Figure S4. Low concentration of acidified nitrite induced cellular starvation for general amino acids.

Strain YMS9 and *cpcA* gene disruptant (*ΔcpcA*) were cultured in MMN medium at 37°C for 18 h, then with or without 1 mM $NaNO_2$ (pH 5.5) for 3 h. Cellular amino acid contents were determined by LC-MS/MS as described in Materials and methods. Values for cellular alanine, asparagine, threonine, methionine, glutamic acid and aspartic acid did not significantly differ. Data are means ± SD of data from three biological replicates (**P* < 0.05, *vs* YMS9 0 mM $NaNO_2$; †*P* < 0.05, *vs ΔcpcA* 1 mM $NaNO_2$ by Student's *t*-test).
